# Haplotype Analysis of *Varroa destructor* and Deformed Wing Virus Using Long Reads

**DOI:** 10.3389/finsc.2021.756886

**Published:** 2021-12-03

**Authors:** Wen Feng Bai, Zhe Guang Lin, Wei Yu Yan, Li Zhen Zhang, Jay D. Evans, Qiang Huang

**Affiliations:** ^1^Honeybee Research Institute, Jiangxi Agricultural University, Nanchang, China; ^2^Jiangxi Province Key Laboratory of Honeybee Biology and Beekeeping, Jiangxi Agricultural University, Nanchang, China; ^3^Apicultural Research Institute, College of Animal Science and Technology, Yangzhou University, Yangzhou, China; ^4^United States Department of Agriculture - Agricultural Research Service (USDA-ARS) Bee Research Laboratory, Beltsville, MD, United States

**Keywords:** haplotype, honey bee, mite, virus, selection, long reads

## Abstract

As a phoretic parasite and virus vector, the mite *Varroa destructor* and the associated Deformed wing virus (DWV) form a lethal combination to the honey bee, *Apis mellifera*. Routine acaricide treatment has been reported to reduce the diversity of mites and select for tolerance against these treatments. Further, different DWV strains face selective pressures when transmitted via mites. In this study, the haplotypes of *Varroa* mites and associated DWV variants were quantified using long reads. A single haplotype dominated the mite mitochondrial gene cytochrome oxidase subunit I, reflecting an ancient bottleneck. However, highly polymorphic genes were present across the mite genome, suggesting the diversity of mites could be actively maintained at a regional level. DWV detected in both mites and honey bees show a dominant variant with only a few low-frequency alternate haplotypes. The relative abundances of DWV haplotypes isolated from honey bees and mites were highly consistent, suggesting that some variants are favored by ongoing selection.

## Introduction

*Varroa destructor* is a an ectoparasitic mite of honey bees, which feeds on honey bee fat bodies and hemocytes (1). During the phoretic stage, mites parasitize adult honey bees and disperse among host individuals and colonies through body contact and host drifting (2, 3). The reproductive phase starts with locating a suitable larval cell. The female mite hides in the brood cell just before it is capped with wax and initiates vitellogenin production. Within the capped cell, the female mite first lays an unfertilized haploid egg that develops into a male, followed by a few fertilized diploid eggs that develop into females (4). The female and male offspring mate within the capped cell, substantially reducing heterozygosity (5). Crossing of mite lineages only occurs when the colony is heavily infected and two or more female mites reproduce within the same brood cell (6). Thus far, the mite mitochondria gene cytochrome c oxidase subunit 1 (*COX1*) was used to identify haplotypes, where only two were found in European honey bees (7).

In addition to the destructive feeding on fat bodies (the most important immune-response tissues in honey bees), mites also transmit several RNA viruses. Among these viruses, Deformed wing virus (DWV), when combined with the mites, critically proliferates in all developmental stages of honey bees (8, 9). DWV originates from European honey bees, but was selected for and vectored by the mite *V. destructor* (10). Currently, the dispersal and titer of DWV in honey bee colonies heavily depends on *V. destructor*. The presence of mites has altered the landscape of DWV strains, in some cases leading to the emergence of dominant strains (11). Acaricides are widely used to control mites, selecting for resistant mite strains and arguably leading to founder effects that reduce mite diversity (12). The aim of this study is to quantify the current diversity of mites and associated DWV. In addition, highly polymorphic loci are selected for haplotype construction. Then the relative abundances of DWV haplotypes are quantified in mites and honey bees to test for competition during the proliferation in a controlled infestation assay.

## Materials and Methods

### Mite Population

Mites (*Varroa destructor*) were collected in four honey bee colonies (*Apis mellifera*), from the experimental apiary in Jiangxi Agricultural University. All colonies were maintained within one apiary. The colonies were 1 year old and had been treated with Amitraz during the summer. Twenty mites were collected from each of four honey bee colonies using the powdered sugar method in Spring (13). The body surface of the mites was rinsed with distilled water and twenty mites of each colony were pooled for RNA extraction using TRIzol. One Pacbio Iso-Seq library was prepared for each RNA pool to sequence in a SMRT cell. In total, four Iso-Seq libraries were prepared and sequenced in four SMRT cells.

### Mite and DWV Haplotype Analysis

The long reads were aligned to the *V. destructor* genome assembly (version *V_des 3.0*) using Minimap2 with default transcript parameters (14). Single nucleotide variants (SNVs) were identified using Longshot with minimal coverage of 20 (15). The SNVs were further annotated with SNPeff (16). SNVs along the contigs were analyzed with Pearson's Chi-Squared test. To analyze the haplotypes of the transcripts, the reads were aligned to the protein coding sequences of the *V. destructor* genome using Minimap2 with default parameters. The SNVs shared in all four colonies were concatenated to form haplotypes using SAM4WebLogo and summarized using Whatshap (15, 17, 18).

RNA reads that could not be aligned to the mite genome were retrieved using Samtools and assigned to microbes using Kraken2 (16, 19). The results were viewed with Krona (20). The reads assigned to Deformed wing virus (strain A) were extracted and aligned to a reference DWV genome (NCBI Genome version GCF000852585.1) with Minimap2. SNVs were predicted using Longshot and the nucleotides at SNV positions were clipped with SAM4WebLogo (13, 17, 21), which were concatenated to form a haplotype for each long read. The distribution of haplotypes among the colonies was analyzed with Pearson's Chi-Squared test, using R (22). Additionally, the reads were aligned to DWV-A (GCF000852585.1), DWV-B (AY251269.2) and DWV-C (CEND01000001.1) in parallel to quantify the relative abundance of DWV variants associated with the mites using Minimap2 (13, 23, 24).

### Transmission of DWV Haplotypes From Mites to Bees

To further study the competition of DWV haplotypes in mites and bees, a controlled mite infestation assay was performed. Second-instar larvae were collected from honey bee frames randomly selected from the colonies in the apiary. The larvae were artificially reared to the pupal stage in 48-well microtiter plates maintained in an incubator, with a temperature and humidity setting of 34°C, 70% RH, respectively (25). *V. destructor* mites were collected from capped cells to minimize any physical damage. During the pupation stage, one mite was transplanted onto each pupa to form a paired infection group (*N* = 10). Pupae without mites served as a control group to assess mortality due to artificial rearing (*N* = 10). Pupae, and their associated mites, were collected 4 days after infestation (N_pupa_ = 5, N_mite_ = 5), as well as the emerged adults (N_pupa_ = 5, N_mite_ = 5). Total RNA was extracted from individual bees and mites with TransZol Up Plus RNA kit (Transgen). cDNA was synthesized using PrimeScript^TM^ RT reagent Kit (Takara), with 1 μl gDNA Eraser L, 2 μl 5 × gDNA Eraser buffer, 5 μl RNase Free H_2_O and 2 μl total RNA incubating at 42°C for 2 min. Additionally, 1 μl PrimeScript RT Enzyme Mix I, 4 μl 5 × PrimeScript Buffer, 1 μl RT Primer Mix and 4 μl RNase Free H_2_O were added to the reaction for 37°C, 15 min, 85°C 5 min. The cDNA was stored at −20°C.

Based on the above SNV analysis of the long reads, the PCR fragments containing the highly dense and variable loci at the 218, 325, 555, and 567th nucleotides were amplified in both mites and bees using customized primers (DWV_h_5': ACGCGCGCGATAATGAGT, DWV_h_3′: GATCTCTGGTTTTGCCTGCAC). A 20 μl amplification reaction system was as follows: 1 μl 5′ primer, 1 μl 3′ primer, 10 μl 2x Tag PCR StarMix, 3 μl DEPC water, 5 μl RNA template. The reaction program consisted of 95°C 5 min, 95°C 10 s, 58°C 30 s, for 40 cycles. The 443 bp PCR product was purified and recovered from an agarose gel using a column DNA back kit (TIANDZ). PCR products were sequenced in both directions using the Illumina Nova 6,000 platform. These paired reads were joined and then aligned to the DWV CDS region using Minimap2 (13). The nucleotides at SNV positions (at 218, 325, 555, and 567th nucleotides) was clipped with SAM4WebLogo to form haplotypes (17). The distribution of haplotypes were analyzed with Pearson's Chi-square test, using R (22). The transmission of haplotypes was visualized using Circlize package in R (26).

## Results

### Dominant Haplotype in Mites

**Figure 1:**
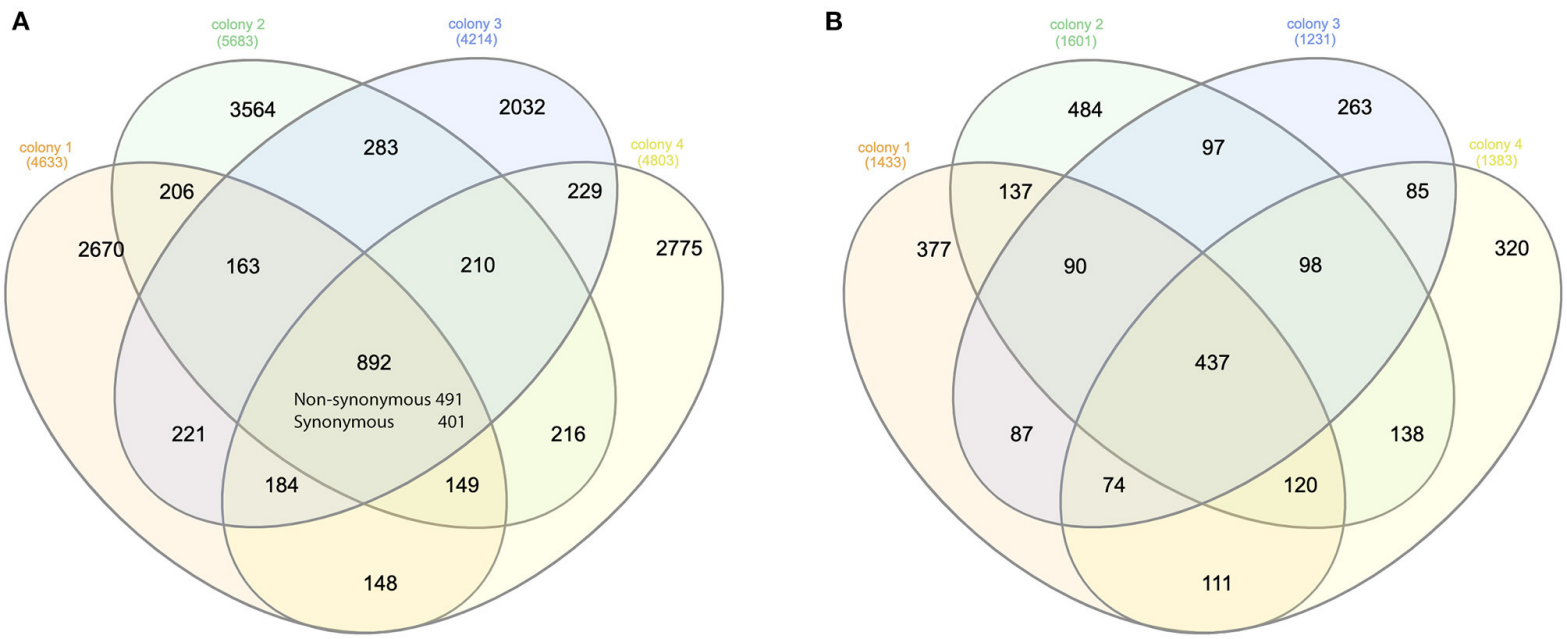
Venn diagram of the SNVs and genes. **(A)** Venn diagram of the synonymous and non-synonymous SNVs among the mites of four honey bee colonies. Overall, 892 SNVs were shared among the four colonies. The number of synonymous SNV was significantly higher than random for each honey bee colony (Pearson's Chi-square test, *P* < 0.001). **(B)** Venn diagram of the genes with non-synonymous SNVs among the mites of four honey bee colonies. Overall, 437 genes were shared in all four colonies, which was again significantly higher than random (Pearson's Chi-square test, *P* < 0.001).

**Figure 2:**
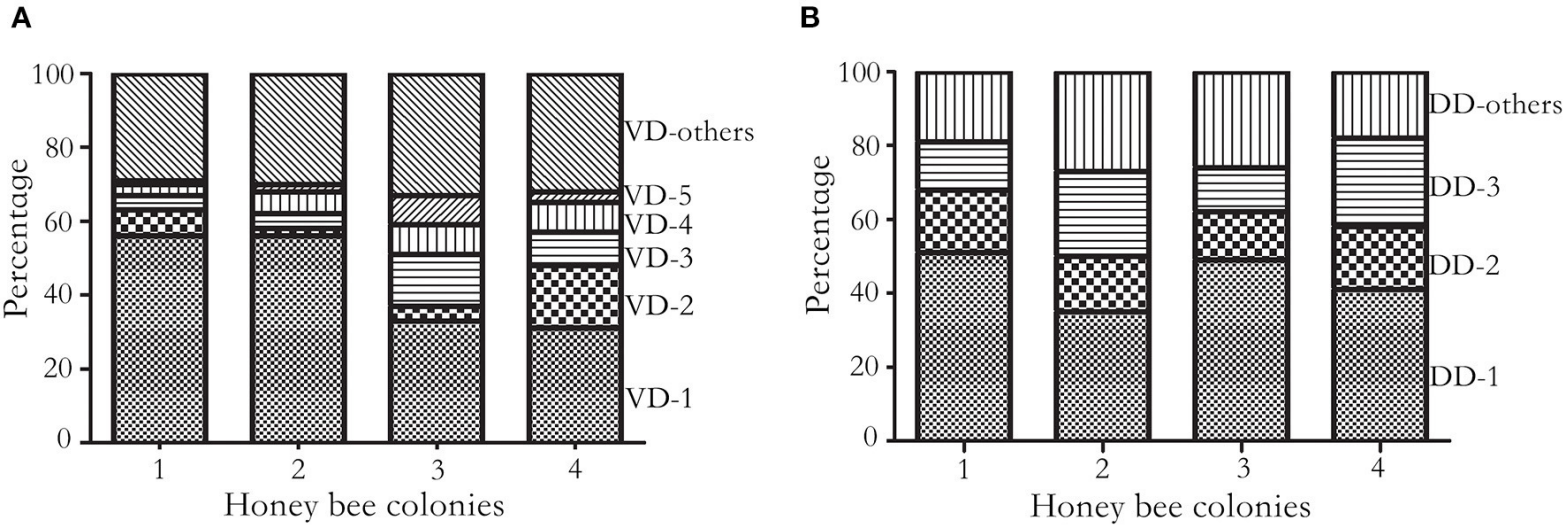
Cumulative frequency histogram of haplotypes. **(A)** Frequency of *V. destructor* haplotypes. Haplotypes were constructed on five SNVs of the most diversified transcript XP_22662619.1 at 4920, 4947, 4957, 4980, and 5076^th^ nucleotide. In total, 30 haplotypes were identified, and 25 haplotypes were shared at least in two colonies. The four colonies shared dominant haplotypes (VD-1 to VD-5) and a few low frequency haplotypes (VD-others). VD represents *V. destructor* dominant haplotype. **(B)** Frequency of DWV haplotypes. Haplotypes were constructed on three SNVs at the 1809, 1938, and 1977^th^ nucleotide. In total, 11 haplotypes were identified, and 8 haplotypes were shared at least in two colonies. Three haplotypes were shared among the four honey bee colonies, which accounted for 70% of total haplotypes. DD represents DWV dominant haplotype.

On average, over 75 million long reads were generated from a SMRT cell, with a mean length of 3.5Kbp (over 150x of the mite transcriptome) (Supplementary Table 1). In total, 4,633, 5,683, 4,214, and 4,803 biallelic SNVs were identified from four honey bee colonies, out of which 892 SNVs were shared (Figure 1). The number of synonymous and non-synonymous SNVs was not significantly different among the four honey bee colonies (Pearson's Chi-square test, *P* > 0.05). On average, 5.25 SNVs were phased (assigned variants to the maternal or paternal chromosome) in a haplotype block (Supplementary Table 2). The mitochondrial genes cytochrome c oxidase subunit I (NP_758874.1), cytochrome c oxidase subunit III (NP_758878.1), and cytochrome b (NP_758884.1) were highly expressed. However, SNVs were not found in either of the above genes, nor for the mitochondrial gene cytochrome c (XP_022658338.1). The transcript activating signal cointegrator 1 complex subunit 3-like (*ASCC3*, XP_022662619.1) showed the highest number of SNVs (146 SNVs) in all four colonies. As 20 mites were pooled, a maximum of 40 haplotypes could be identified from this gene in each colony. In theory, 6 biallelic SNVs (2^6^ = 64) were sufficient to distinguish between the 40 haplotypes. Two SNVs at the 3' end of the transcript XP_022662619.1, with the highest coverage, were used as an anchor to construct haplotypes. One SNV was added at a time, until the observed number of haplotypes was less than the expected ones. In total, 30 haplotypes were identified using 5 SNVs and 12 haplotypes were shared among the colonies (Supplementary Figure 1). Surprisingly, a dominant haplotype (VD-1) was found in all four colonies (Figure 2A).

### Dominant Haplotype of Deformed Wing Virus in Mites

On average, 525,922 reads aligned to microbes associated with mites. Among the DWV variants, 95.41 ± 2.02% (Mean ± SD) reads were assigned to DWV-A, which was orders of magnitude higher than DWV-B (4.58 ± 2.01%) and DWV-C (0.02 ± 0.01%). In total, 381 SNVs were identified from DWV, among which 11 SNVs were commonly shared among the four colonies (at 218, 325, 555, 567, 615, 693, 780, 1809, 1938, 1977, and 8676^th^ nucleotide, respectively) and 266 SNVs were colony specific. The proportion of shared SNVs was significantly higher in mites compared with DWV along the genome (Pearson's Chi-square test, *P* < 0.05). Three commonly shared SNVs at the 1809, 1938, and 1977^th^ nucleotides were used to construct the haplotypes. In total, 11 haplotypes were identified, and 3 haplotypes dominated over 70% of total haplotype counts in all four colonies (Figure 2B; Supplementary Figure 2).

### Transmission of DWV Haplotype From Mites to Bees

**Figure 3:**
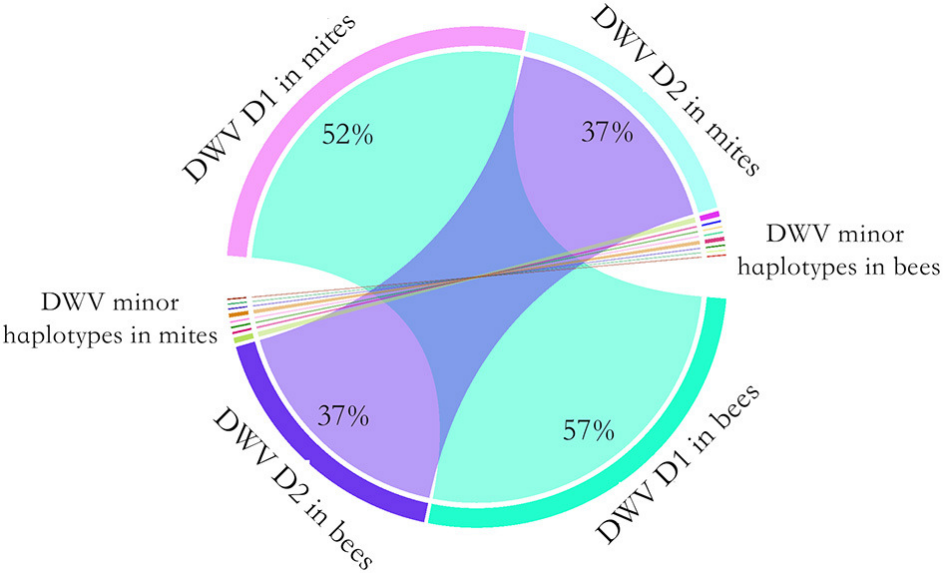
Chord diagram of the DWV haplotype transmission from the mites to honey bees. To quantify the transmission of DWV haplotypes from mites to bees, a controlled mite infestation assay was performed. The larvae were artificially reared in 48-well microtiter plates and a female mite was transplanted to each of the larvae. Then DWV haplotypes was quantified for each bee-mite pair. Two DWV haplotypes dominated in both bee and mites. The dominant haplotype 1 (D1) accounted for 52% of observed haplotypes in mite, which accounted for 57% of observed haplotypes in bees. The dominant haplotype 2 (D2) accounted for 37% of observed haplotypes in mite, which also accounted for 37% of observed haplotypes in bees. A few minor haplotypes were also observed in bees and mites. Overall, the relative haplotype abundance of DWV in bees was highly consistent with the mites (Fisher's exact test, *P* < 0.001).

To determine whether the dominant DWV haplotypes in bees originated from high virus abundance in mites, or by viral competition during proliferation, a controlled mite infestation assay was performed. As noted above, for this experiment larvae were reared in a laboratory incubatory until pupation. All larvae survived this incubation. Then, each experimental pupal sample was paired with a *Varroa* mite. In the control group (no mite), DWV was not detected. In the treatment groups, 2.2 million PCR fragments were sequenced. The 218, 325, 555, and 567^th^ nucleotides were identified as having the highest diversity, on average, in the DWV genome. Surprisingly, two haplotypes accounted for 95% of DWV in both the mites and the bees. By analyzing the paired pupae and mite, the relative abundance of the DWV haplotypes in mites and bees was consistent, a result that deviated significantly from random (Fisher's exact test, *P* < 0.001) (Figure 3).

## Discussion

*V. destructor* is a native ectoparasite of the Asian honey bee that has successfully established infestation in European honey bee colonies. The impact of *V. destructor* infestation on the native host is minor, as the bees can effectively inhibit mite reproduction (27). However, the novel host lacks this acquired defense and the number of the mites infesting colonies can increase by orders of magnitudes, often leading to the collapse of the colony (28–30). Previously, 6 haplotypes of *V. destructor* were identified based on a 458 bp DNA fragment of cytochrome c oxidase subunit 1 (*COX1*), with one haplotype per geographic location (7). Two (Korea and Japan haplotypes) out of these 6 haplotypes were found parasitizing the novel host honey bees and the remaining 4 haplotypes were confined to the native host honey bees (7). However, within the Korea and Japan haplotypes, mite haplotypes cannot be further refined using the *COX1* gene. Instead, a highly polymorphic nuclear gene is required. In our data, SNVs were not found in the *COX1* gene. Further, the *COX1* gene perfectly aligned to the Korean haplotype sequence, suggesting all 80 studied mites originated from this lineage (31). This haplotype has spread to, and thrived in, novel host honey bees while also maintaining reproductive potential in the native host honey bees (27, 32). In our study, we identified a highly polymorphic gene, *ASCC3*, which can serve as an alternate candidate gene for fine-scale population genetic studies in these mites.

Although current diversity in mite populations is low, variation among colonies and apiaries has been reported (33, 34). In our data, the small number of SNVs suggests low standing genetic diversity. Colony-level variation exists and the overall selective pressure on mites remains similar at the colony level, as suggested by the number of synonymous and non-synonymous SNVs. Additionally, the Korea haplotype may have switched back to the native host, boosting mite diversity and facilitating the exchange of viruses (33, 35, 36). DWV is transmitted from queens to offspring, by feeding on contaminated food, and via feeding by mites. In the absence of mites, infection levels of DWV were generally low and didn't present the indicative symptom of deformed wings (37). In natural conditions, DWV was found in pollen, feces and flowers, facilitating horizontal transmission among pollinators, as well as to insect predators (38–42). Spillover of DWV among pollinators could enhance mutation and recombination events. Multiple transmission routes could lead to increased DWV diversity (43–45), perhaps helping to explain why observed DWV diversity is higher than that of mites. In our data, several bee viruses were associated with *V. destructor* but DWV was the most abundant of these. Using amplified fragments of DWV, haplotype variance was found among bee colonies (46, 47). The diversity between individuals remained high and 286 divergent nucleotides were identified from DWV isolated from bees (48). In our data, 381 SNVs were identified from DWV isolated from mites and numerous haplotypes were found from each colony. Less than 5% of the SNVs were commonly shared among colonies and the distribution of haplotypes was significantly different among the colonies. Interestingly, we found the relative abundance of DWV was highly consistent in paired mites and bees. Even though the transmission of DWV between honey bees and mites is reciprocal and the mites may contain bee cells, the impacts on haplotype structure are minor as long as the dominant haplotypes are circulating in local population. Overall, our results suggest minor competition among the haplotypes during the proliferation within the selected mite—honey bee pairs. Our study is limited by using randomly collected phoretic mites, different gene expression patterns may be identified using other life stages.

## Data Availability Statement

The datasets presented in this study can be found in online repositories. The names of the repository/repositories and accession number(s) can be found here: NCBI [accession: PRJNA726354].

## Author Contributions

WB conducted controlled mite infestation analysis. QH conceived the project. WB, ZGL, LZZ, WYY, JE, and QH wrote the manuscript. All authors contributed to the article and approved the submitted version.

## Funding

This project was funded by Science and Technology Program Project of Jiangxi Province 20202BBFL63028 (QH), initiation package of Jiangxi Agricultural University 050014/923230722 (QH), National Natural Science Foundation of China 31902220 (ZGL), and the earmarked fund for Jiangxi Agriculture Research System JXARS-14 (WYY). Natural Science Foundation of Jiangxi Province 20192BAB214022 (LZZ). Supported by USDA National Institute of Food and Agriculture (NIFA) grant 2017-06481 (JE).

## Conflict of Interest

The authors declare that the research was conducted in the absence of any commercial or financial relationships that could be construed as a potential conflict of interest.

## Publisher's Note

All claims expressed in this article are solely those of the authors and do not necessarily represent those of their affiliated organizations, or those of the publisher, the editors and the reviewers. Any product that may be evaluated in this article, or claim that may be made by its manufacturer, is not guaranteed or endorsed by the publisher.
