# Supplementary material for: Haplotype Analysis of Varroa destructor and Deformed Wing Virus Using Long Reads
Source: Front Insect Sci. 2021 Dec 3;1:756886. doi: 10.3389/finsc.2021.756886 (PMC10926369; doi:10.3389/finsc.2021.756886)
Supplement: Supplementary file 1 [file Data_Sheet_1.PDF]

Haplotype analysis of *Varroa destructor* and *Deformed wing virus* using long reads

Wen Feng Bai[1,2], Zhe Guang Lin[3], Wei Yu Yan[1,2], Li Zhen Zhang[1,2], Jay D. Evans[4], Qiang Huang*[1,2]

[1] Honeybee Research Institute, Jiangxi Agricultural University, Zhimin Ave. 1101, Nanchang, 330045, China.

[2] Jiangxi Province Key laboratory of Honeybee Biology and Beekeeping, Jiangxi Agricultural University, Zhimin Ave. 1101, Nanchang, 330045, China.

[3]Apicultural Research Institute, College of Animal Science and Technology, Yangzhou University, South University AVE. 88, Yangzhou, 225009, China.

[4] USDA-ARS Bee Research Laboratory, BARC-East Building 306, Beltsville, Maryland, 20705, USA.

*for correspondence: qiang-huang@live.com (Q.H.)

Results

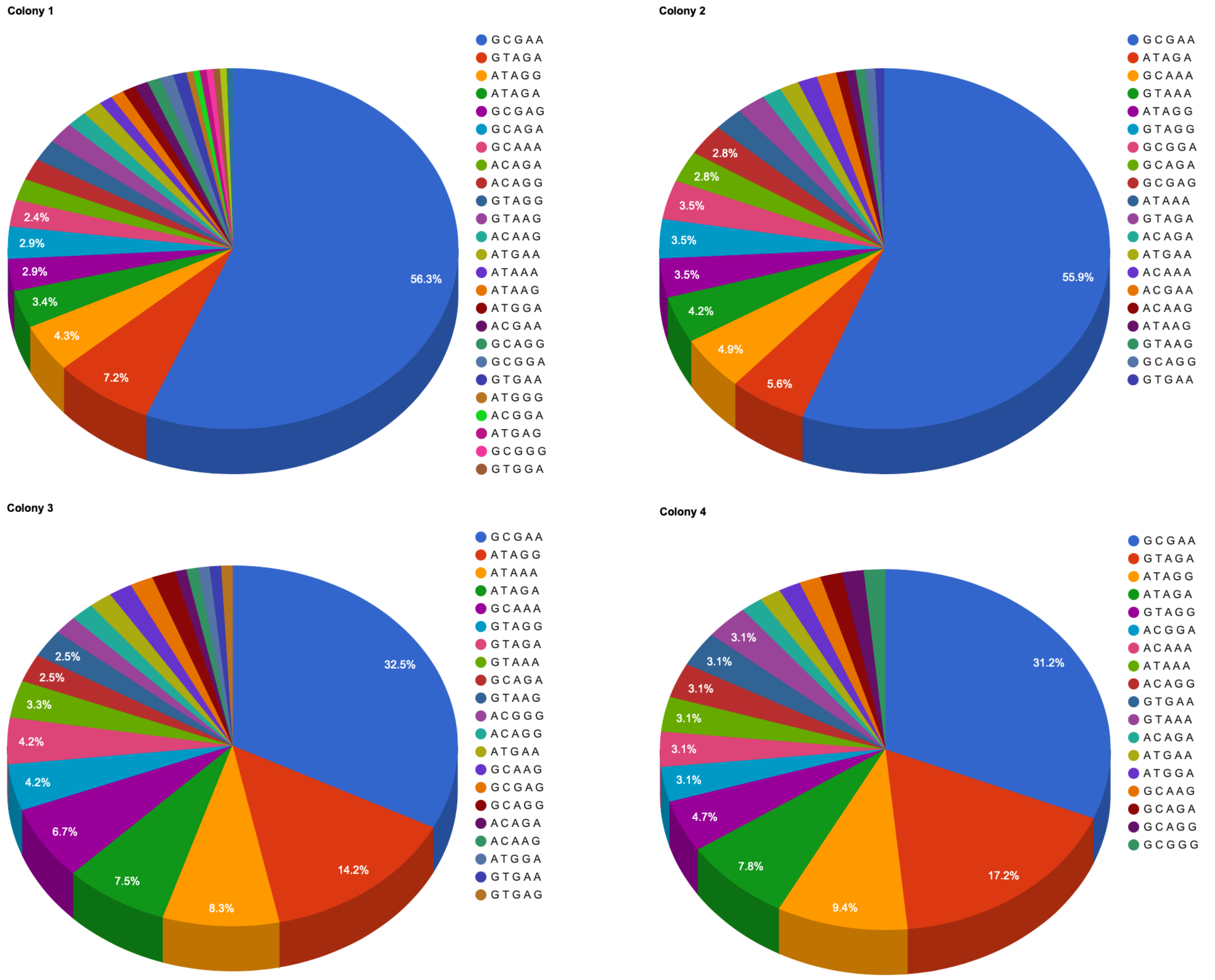

Figure S1 Pie chart of the haplotype variants across mites. Haplotypes were constructed on five SNVs of the most diversified transcript XP_22662619.1 at 4920th, 4947th, 4957th, 4980th and 5076th nucleotide. In total, 30 haplotypes were identified, where 25 haplotypes were shared at least in two colonies and 12 haplotypes were shared in four colonies. Additionally, a common haplotype dominant all four colonies along with a few low frequency haplotypes.

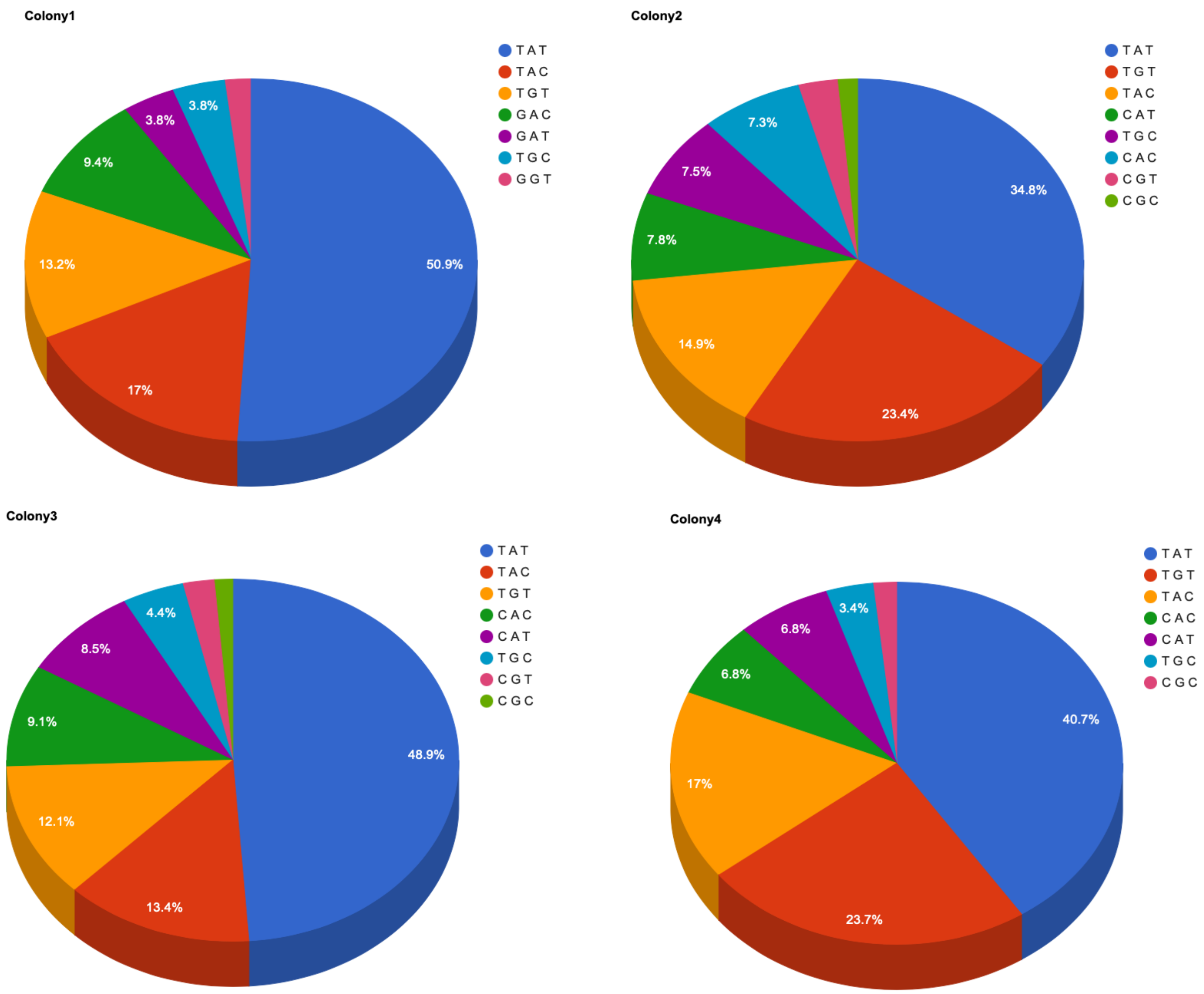

Figure S2 Pie chart of haplotype components for the DWV. Haplotypes were constructed on three SNVs within the viral particle region at the 1809th and 1938th and 1977th nucleotide. In total, 11 haplotypes were identified, and 8 haplotypes were shared at least in two colonies. The four colonies shared 4 common dominant haplotypes accounting for 75% of total haplotypes.

Table S1 statistics of the RNA reads of four pools of *V. destructor* and the genome annotation of coding sequences.

Sequenced reads	colony 1	colony 2	colony 3	colony 4
#reads aligned to mites genome	88,648,370	60,877,945	104,178,382	74,063,517
#reads aligned to microbes	229,866	1,301,937	407,098	164,787

Table S2 Constructed haplotypes from the mites' transcripts among the honey bee colonies

	Colony 1	Colony 2	Colony 3	Colony 4	Among colonies variance
Total SNVs #	6,329	6,577	7,335	6,451	$\chi^2 = 44.8$, df = 3, $P < 0.001$
Phased SNVs #	5,175	5,112	5,915	5,240	$\chi^2 = 37.8$, df = 3, $P < 0.001$
Average block size [SNVs]	5	5	6	5	$\chi^2 = 0.06$, df = 3, $P = 0.9$
Average block length [bps]	907	910	927	987	$\chi^2 = 2.19$, df = 3, $P = 0.5$
